# ASO Author Reflections: Advanced Imaging Allows Better Detection of Peritoneal Metastases

**DOI:** 10.1245/s10434-018-7083-4

**Published:** 2018-11-26

**Authors:** Nina Roelie Sluiter, Jurriaan Benjamin Tuynman

**Affiliations:** Department of Surgery, Cancer Center Amsterdam, Amsterdam UMC, Vrije Universiteit Amsterdam, Amsterdam, The Netherlands

## Past

Peritoneal metastases (PM) are diagnosed in up to 30% of patients with colorectal cancer, especially in patients with T4 disease.^1^ Cytoreduction and hyperthermic intraperitoneal chemotherapy (HIPEC) improve the survival of patients with colorectal PM, resulting in an overall survival of 56 months in selected patients with limited peritoneal disease.^2^ Unfortunately, PM are often missed during elective primary tumor resection. Second, during follow-up after initial curative treatment, PM are mostly diagnosed in an advanced stage. Current diagnostic imaging modalities such as positron emission tomography/computed tomography (PET/CT) have a detection limit of 5 mm and the multiple small peritoneal lesions are frequently missed.^3^ The presence of advanced intra-abdominal disease such as omental cake can be easily visualized, but curative options in this stage are limited. Since cytoreduction and HIPEC results in good survival in patients with limited peritoneal disease, accompanied by a low morbidity due to the limited amount of resections, extensive effort should be made to improve early diagnosis of PM.

## Present

Advanced imaging could improve the detection of PM during elective colorectal cancer resection and diagnostic laparoscopy. Narrow-band imaging (NBI), near-infrared indocyanine green fluorescence imaging (NIR-ICG), and spray-dye chromoendoscopy (SDCE) were prospectively compared with white-light imaging for the detection of PM in 28 patients.^4^ NBI substantially increased sensitivity from 80.0% with white light to 96.0% (*p* = 0.008), without loss of specificity (73.1% vs. 74.8%, *p* = 0.804). NIR-ICG and SDCE were not considered of value: NIR-ICG did not result in fluorescent PM and SDCE did not visualize the whole peritoneum.

NBI is not only a promising and safe method but is also practical, time efficient, and does not require extra costs. Its main implication could be early detection of PM during evaluation of the peritoneum at the time of primary tumor resection. This allows early referral to specialized HIPEC centers, which is key for oncologic outcome. Second, NBI could help determine feasibility of a complete cytoreduction prior to HIPEC. Third, improved visualization of PM may optimize cytoreduction.

## Future

NBI is a practical technique that is available on most laparoscopic systems, facilitating quick translation into clinical practice. Fluorescence with antibody-coupled ICG is a promising method but is currently not widely available and requires further research prior to clinical implementation. Issues to be addressed include identification of optimal antibody targets (vascular endothelial growth factor or carcinoembryonic antigen) and cost-effectiveness. Furthermore, investigation of the value of advanced imaging in terms of oncologic outcomes is pivotal, warranting prospective randomization between techniques. Currently, a trial evaluating second-look diagnostic laparoscopy after T4 colorectal cancer resection aims for detection of PM at a clinically occult stage, which is expected to translate into survival benefit (ClinicalTrials.gov: NCT03413254). To further improve survival of patients with colorectal PM, early diagnosis by advanced (epi-)genetic analyses in peripheral blood as liquid biopsies has been studied.^5^ Together with advanced intraoperative tumor detection, this could allow for cytoreduction and HIPEC in an early and curable stage of peritoneal disease, and, consequently, improve oncologic outcomes.
